# A hybrid vocal fold phonatory platform for pediatric phonation modeling

**DOI:** 10.3389/fbioe.2025.1699406

**Published:** 2026-01-21

**Authors:** Leila Donyaparastlivari, Rishi Kuriakose, Mohaddeseh Mohammadi, Ayda Pourmostafa, Daniel Li, Scott L. Thomson, Chen Shen, Amir K. Miri

**Affiliations:** 1 Department of Mechanical and Industrial Engineering, New Jersey Institute of Technology, Newark, NJ, United States; 2 Department of Biomedical Engineering, New Jersey Institute of Technology, Newark, NJ, United States; 3 Department of Mechanical and Civil Engineering, Brigham Young University-Idaho, Rexburg, ID, United States; 4 Department of Mechanical Engineering, Rowan University, Glassboro, NJ, United States

**Keywords:** vocal folds, self-sustained oscillations, silicone, gelatin methacryloyl, biomechanics

## Abstract

Understanding pediatric phonation requires models that capture the biomechanical properties and dynamic airflow interactions of vocal folds. While synthetic vocal fold models have advanced the study of airflow–structure interactions in phonation, they cannot incorporate biologically relevant components such as hydrogels or human-derived cells. We developed a hybrid vocal fold phonatory platform that integrates a natural hydrogel with a silicone-based synthetic framework to address this limitation, enabling biomechanical fidelity and biological relevance. We adapted and downscaled a human vocal fold model (EPI) to replicate the dimensions of infant vocal folds. Using silicone elastomers and gelatin-silicone composites, we fabricated infant-scale replicas that mimic native tissue. Our results demonstrate that the material properties and geometrical scaling significantly affect vibratory behavior and acoustic output. Size reduction aligns with pediatric anatomical dimensions and minimizes the cell volume required for future biologically active models. This platform offers a scalable and bio-integrative approach for studying pediatric phonation, with potential applications in voice biomechanics, developmental vocal fold pathology, and tissue engineering.

## 1 Introduction

The primary physiological function of the vocal folds - a pair of soft connective tissues - involves generating audible air pulses for their self-sustained oscillations, a passive acoustic interaction between airflow and the tissue (Thomson, 2024; Beeck et al., 2022; Kreiman and Lee, 2025; Guasch, 2025). Infant vocal folds are structurally and functionally distinct from those of adults, reflecting the unique biomechanical demands of early phonation and airway protection (Hartnick et al., 2005). Neonatal vocal folds lack the developed layered structure seen in adults; the lamina propria is underdeveloped, with a high water content and limited differentiation between superficial, intermediate, and deep layers (Hartnick et al., 2005). Infant vocal folds are more vulnerable to iatrogenic and inflammatory insults. Common conditions include laryngomalacia (Leung and Cho, 1999), subglottic stenosis (Pasick et al., 2022), and congenital webs (Filauro et al., 2020), which can impair airway function and phonation. The development of pediatric-appropriate vocal fold models has lagged behind adult-focused studies in the literature.

Geometrical metrics, such as depth, thickness, and length, as well as the mechanical properties of the materials, such as stiffness, are commonly optimized for mimicking vocal fold dynamics during phonation (Riede et al., 2024; Rubin et al., 2025; Li et al., 2016; Lehoux et al., 2021; Romo et al., 2025). Engineered models are crucial in advancing our understanding of voice production and its clinical pathologies by enabling the controlled simulation of abnormal phonatory conditions (mainly published for adult voice disorders). For example, excessive loading in the laryngeal muscles may increase laryngeal tensile forces (Hsiao et al., 2001) and viscoelastic properties of the vocal folds (McKenna et al., 2019). Airflow-based models allow the estimation of the physical parameters related to vocal fold self-sustained oscillations, such as the fundamental frequency of phonation (Schoder et al., 2024). Viscoelastic synthetic vocal fold models have successfully created flow-induced self-sustained oscillations and audible sound sources (Thomson, 2024). However, such models cannot be easily translated into biological studies on the role of biological factors in phonation.

Rigid-walled replicas, made of silicone rubber, were used to study how geometric parameters influence trans-laryngeal pressure drop and recovery, airflow resistance, and glottal jet dynamics [e.g., (Gühring et al., 2024; Tur et al., 2023; Zhang et al., 2024; Guasch et al., 2022)]. With the achievement of self-oscillating single-layer silicone-based replicas [e.g., (Thomson et al., 2005; Hasegawa et al., 2024; Zhao and Singh, 2023; Chan and Titze, 1998; Kimura and Tayama, 2013)], interest has shifted to multilayer replicas (Tur et al., 2023; Greenwood and Thomson, 2021; Tur et al., 2024; Kaufmann et al., 2024), making them more biocompatible for cell loading (Svistushkin et al., 2023; Tindell et al., 2022). The experimental research has examined the vocal fold of vibratory patterns and the resulting sound propagation to understand the fluid-structure interactions (FSI). These FSI studies uncover vocal fold responses under fixed posturing and specific biomechanical properties.

Ecoflex® has been widely used for making vocal fold replicas. Modifying the internal pressure of fluid-filled cavities in membrane-based models (Zhang et al., 2024; Zhang, 2016), altering the chemical composition or processing methods of different polymers used in 3D-molded replicas (Michaud-Dorko et al., 2024; Michaud-Dorko et al., 2025), integrating fibrous reinforcements (Gühring et al., 2024; Treinkman and Johns, 2024), or introducing localized surface mechanical variations (Guasch et al., 2022; Cha and Thibeault, 2025), can all influence the oscillatory behavior of artificial vocal folds. These variables can be critical of the supraglottal/subglottal pressure (Zhang, 2016; Risse et al., 2024; Tian et al., 2014; Chan et al., 2025; Luizard and Pelorson, 2017), sound spectrum (Riede et al., 2024; Gühring et al., 2024; Tur et al., 2024; Michaud-Dorko et al., 2024; Risse et al., 2024), glottal jet dynamics (Risse et al., 2024), vocal-fold closure (Zhang, 2016), and surface motion. In parallel, over the past decades, the integration of computational models has enhanced the examination of vocal fold dynamics through simulation (Schoder et al., 2024; Guasch et al., 2024). Using mass-spring-damper vibration models to simulate vocal folds to study their natural mode frequencies (Guasch et al., 2024; Lee et al., 2024; Serry et al., 2023; Döllinger et al., 2023; Loumeaud et al., 2024; Jiang et al., 2024), FSI (Jiang et al., 2024; Deng and Peterson, 2024; Zakerzadeh et al., 2025; Sadeghi et al., 2019), or finite element analysis (FEA) simulations [e.g., (Michaud-Dorko et al., 2024; Zhang, 2016; Jiang et al., 2024)]. The physiological function of the vocal folds involves tensile, shear, and bending stiffness during self-sustained oscillations and quasi-static tension affecting the regulation of their natural vibration frequencies (Luizard and Pelorson, 2017; Lee et al., 2024; Khan, 2025). The airflow from the lungs through the trachea and into the larynx, passing between the vocal folds. The air propagation creates pressure differences that can cause the folds to oscillate. This describes the non-linear and anisotropic behavior of the tissues at the macroscale level, which can change their eigenmodes, onset fundamental frequency (Luizard and Pelorson, 2017; Jiang et al., 2024; Deng and Peterson, 2024; Kafkas, 2024; Chung et al., 2024), opening and closing pattern (Döllinger et al., 2023; Jiang et al., 2024; Deng and Peterson, 2024), airflow rate (Michaud-Dorko et al., 2024; Döllinger et al., 2023), and sound pressure level (Zhang, 2016; Miri, 2014).

Three established vocal fold geometries to simulate human phonation are: Epithelial (EPI) (Murray and Thomson, 2012; Taylor and Thomson, 2022), MRI (Ekström et al., 2025; Azzouz et al., 2025), and M5 (Khan, 2025; Švec et al., 2025). The M5 model integrates FSI computations and mimics how airflows influence vocal fold behavior (Perrine and Scherer, 2023). For MRI designs, high-resolution MRI scans are obtained to capture the anatomical details of the vocal folds (Wilson, 2024), and the EPI model integrates multiple layers, each mimicking the biological components of real vocal folds (Michaud-Dorko et al., 2024). Although significant progress has been made in material selection (Michaud-Dorko et al., 2024; Terzolo et al., 2022), structural refinement (Terzolo et al., 2022), and mechanical tuning (Tur et al., 2023; Terzolo et al., 2022), challenges remain in the capability of cell-laden mimetic phonatory systems.

In this study, we introduced an experimental platform based on a single-layered EPI design and downscaled to match the typical dimensions of young children’s and infants’ vocal folds. We used silicone rubber to make flow-induced oscillations and regulate phonatory parameters. We measured the threshold of phonation pressure, fundamental frequency of phonation, vocal folds trajectories (VFT), high-speed video kymography (VKG), and glottal optical flow waveform (GOFW). Then, we added a volume of natural gelatin-based hydrogel to encapsulate biological cells and condition via self-sustained oscillations.

This voice platform enables quantitative modeling of biophysical factors that regulate phonatory function, particularly the fundamental frequency of phonation. The system facilitates investigation of how variations in vocal fold tissue stiffness, subglottal (lung-driven) airflow pressure, and oscillation amplitude influence the behavior of encapsulated cells within a physiologically relevant microenvironment. Evaluating vocal fold cell responses in the presence of therapeutic compounds establishes a physiomimetic platform for drug screening under dynamic mechanical conditions. Rooted in its miniaturized design and potential scalability, this model can also be adapted to compare phonatory parameters across animal models and human systems, supporting translational studies of vocal fold biology and pathology.

## 2 Materials and methods

### 2.1 Airflow set-up

**FIGURE 1:**
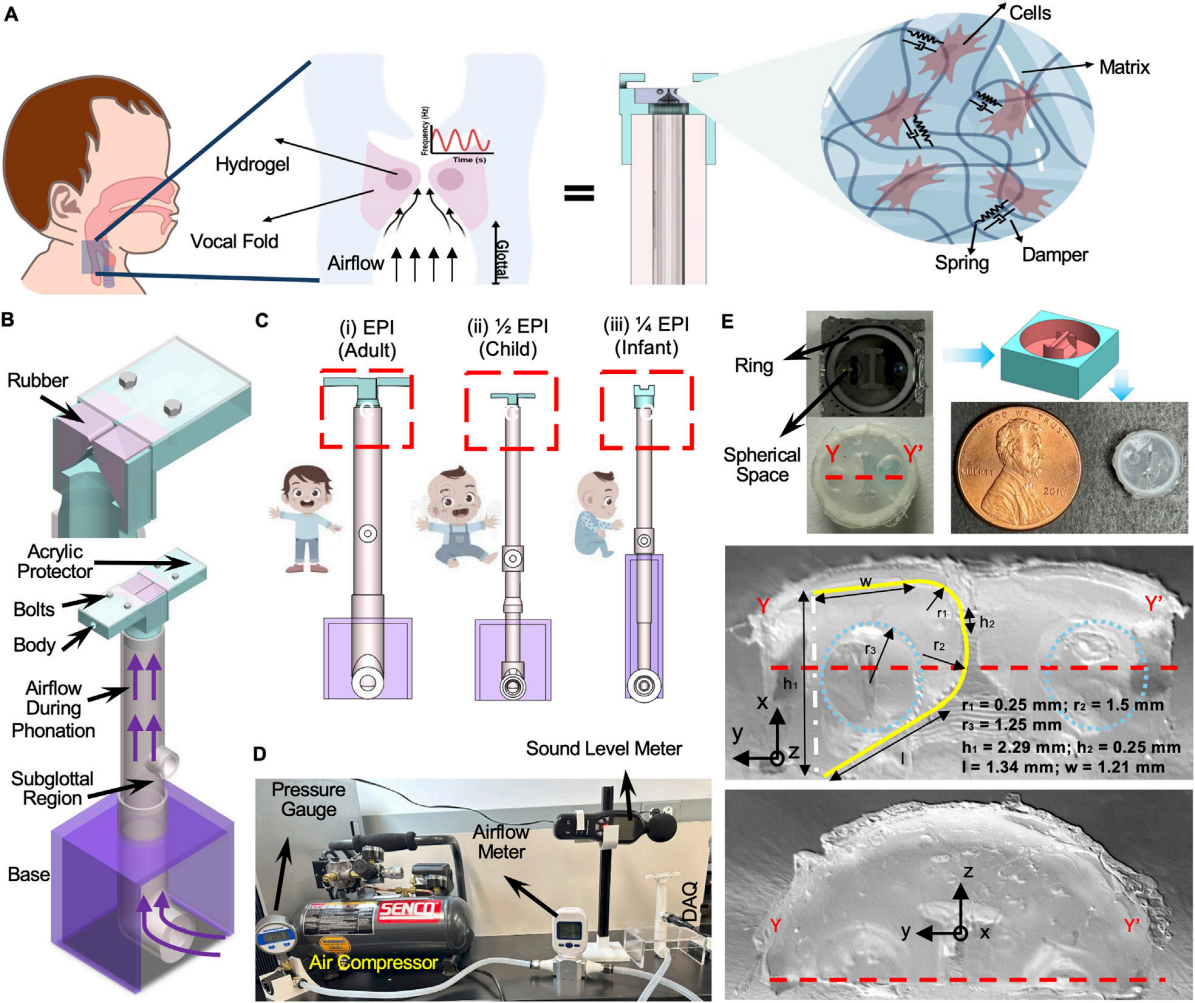
**(A)** Miniaturized (EPI 1:4) vocal fold replica mimicking infant phonation. Airflow induces oscillation in a silicone structure with the hydrogel, where 3T3 cells are embedded. The zoomed-in view illustrates the viscoelastic microenvironment modeled by spring-damper elements, representing cell–matrix interactions under vibration. **(B)** Structural diagrams of the EPI and EPI 1:2 larynx replicas, showing key components including the rubber-based vocal folds, acrylic support, bolts, subglottal chamber, and airflow pathway. **(C)** Schematic comparison of model sizes corresponding to different developmental stages—adult (EPI), child (EPI 1:2), and infant (EPI 1:4)—illustrated with age-matched cartoon figures. **(D)** Experimental phonation setup including a compressor, pressure gauge, airflow meter, sound level meter, and DAQ system. **(E)** EPI 1:4 replica with cross-sectional imaging (A–A′) displaying tissue placement (ring and sphere), geometric measurements, and spatial coordinate axes (X, Y, Z). The scaled-down models are designed to mimic age-specific vocal fold mechanics.

The set-up includes a pair of silicone rubber replicas affixed to a custom-designed body as shown in Figure 1. The two replicas were placed in lateral contact, resembling the collision contact between the human vocal folds. The glottis distance was considered 0.3 mm. The bioreactor body with a tubing system was mounted on a Plexiglass base to balance the structure vertically or horizontally. The EPI model was examined at three ratios: 1, 1/2, and 1/4, as shown in Figures 1B-C. The custom-designed bodies were proportionally scaled to match the dimensions of their respective vocal fold models. The bioreactor assembly was interfaced with a compressor (Senor air compressor, PC 1010), a pressure transducer (100PSI Pressure Transducer), a pressure gauge (IP54 Stainless Steel), and an airflow meter (MF5700 series airflow meter). The outlet diameter of each subglottal region in the EPI model is scaled down to match the size of the bioreactor body, while the inlet diameter remains unchanged to accommodate the fixed tubing size. Transparent industrial plastic tubing connects the air compressor to a pressure gauge, which displays the internal air pressure. This tubing then connects to an airflow meter that measures the flow rate in liters per minute. The tube outlet is connected to the subglottal inlet to direct airflow into the vocal fold replicas. A hole is incorporated into each subglottal region to install a pressure transducer, positioned 10 cm from the vocal replicas as referenced in Cha and Thibeault, (2025). Push-to-connect fittings are used at all junctions to prevent air leakage (Figure 1D).

### 2.2 Synthetic model

A 3D computer-aided design (CAD) model of the vocal fold replica was developed using SolidWorks 2024 for our preliminary data. We selected the EPI model for its scalability and ideal shape for simulating phonation (Bouvet et al., 2020). The dimensions of the EPI mold were scaled down to 1/2 and 1/4 using the scaling feature in SolidWorks. The EPI mold was printed using a commercial 3D printer (Adventure 5M), while the 1/2 and 1/4 scaled EPI molds were produced using another printer model (Stratasys F170 3D laser machine). After printing, the molds were prepared for use; the EPI mold required manual removal of support structures. The molds produced by the other printer were submerged in a water solution for approximately 4 h before they could be used. The tissue housing for EPI and 1/2EPI was designed and fabricated using a 3D printer to secure the vocal fold replicas during phonation. The design for the housing and vocal replicas of the down-scaled EPI is different from the two designs. As depicted in Figure 1E, the CAD mold is shown in pink and blue. The molds are filled with silicone rubber polymer, then a 3D-printed ring is added to hold the replicas under high pressure. The two spherical pearls are added on both sides to inject different materials and observe the effectiveness of the composite materials on the fundamental frequency. Synthetic vocal fold replicas are fabricated using two materials with varying stiffness properties according to the procedure detailed in Supplementary Figures S1, 2. Two-part silicone rubber and single-component silicone thinner were utilized to create replicas. The components, including part A, part B, and silicone thinner, were mixed in a mass ratio of 1:1:x, where x varied as 0, 0.5, 1, and 1.5 to achieve viscoelastic properties like those of cadaveric human vocal fold covers. A vacuum pump was employed to evacuate the mixture and eliminate air bubbles. The mixture was then poured into the molds and cured in an oven for 1 hour (at 60 °C). After curing, the replica was carefully removed from the mold, and any excess material was trimmed.

### 2.3 Phonatory functions

To ensure physical consistency across scales, airflow rate and material modulus were tuned according to nondimensional scaling criteria derived from Reynolds and Womersley similarity, maintaining dynamic similarity between adult and pediatric-sized replicas. While the models differ in absolute dimensions, their vibratory behavior is governed by equivalent dimensionless relationships among geometry, material stiffness, and flow pressure.

The design shown in Figure 1B includes two main parts: the body and the subglottal area. The body of the bioreactor consists of vocal fold pairs and acrylic sheets with bolts and nuts to hold replicas inside the body during phonation. A laser-cut Plexiglass base is added to keep the structure during the experiment. Dynamic supraglottal pressure is monitored using a digital microphone (SLM-25 professional sound level meter) ∼10 cm above the folds. Data from the microphone was processed using the software (NoiseLogger Communication Tool). The embedded Omega pressure transducer is connected to the DAQ (data acquisition system) to extract the data through a customized code written in MATLAB R2024a.

### 2.4 Mechanical characterization

The tensile elastic modulus is measured using an Instron testing machine. The three replicas of each of the four different ratios of polymers are prepared according to the ASTM type V standard. The samples are placed between two grips, and tensile strain is applied incrementally up to 100% to assess their mechanical properties. A rheometer measures the shear elastic and loss moduli of the material groups. All samples are made with a diameter of 25 mm and a thickness of 1,000 μm to examine. The distance between the two plates will be carefully calibrated before conducting the tests. The shear moduli of the samples will be determined across a frequency range of 0.5–100 Hz and a strain range of 1%.

### 2.5 Hydrogel fabrication

This simplified model has two regions: a homogeneous viscoelastic rubber with isotropic behavior and a poroelastic hydrogel with isotropic deformation response. The hydrogel part was synthesized following the literature. Gelatin methacryloyl (GelMA) was prepared following a modified version of previously established protocols 65, 66.10% w/v porcine skin gelatin (CAS 9000–70–8; Sigma-Aldrich). 10% (w/v) porcine skin gelatin (CAS 9000–70–8; Sigma-Aldrich, St. Louis, MO, USA) was dissolved in Dulbecco’s phosphate-buffered saline (DPBS; Sigma-Aldrich) within a 100 mL glass flask. To minimize evaporation, the flask was covered during dissolution and stirred continuously with a magnetic stir bar at 60 °C on a hot plate until the solution became fully homogeneous, which typically required about 1 hour. Once dissolution was complete, 5 mL of methacrylic anhydride (CAS 760–93–0; Sigma-Aldrich) was gradually introduced into the solution while reducing the temperature to 50 °C. The mixture was allowed to react under constant stirring for an additional hour. To terminate the reaction, a fivefold volume of pre-warmed DPBS was added. The resulting GelMA solution was subsequently purified by dialysis against deionized water using dialysis tubing with a 12–14 kDa molecular weight cutoff for 1 week to remove unreacted methacrylic anhydride. Following dialysis, the product was aliquoted into centrifuge tubes and preserved at −80 °C for future use. On the day of the experiment, the lyophilized GelMA was dissolved in DPBS and mixed with lithium phenyl-2,4,6-trimethylbenzoylphosphinate (CAS 85073-19-4; Sigma-Aldrich) 0.1% w/v, as our photo-initiator. Within each vocal fold replica, both high- and low-volume hydrogel formulations were incorporated. Gelatin constructs were allowed to gel by refrigeration for 1 hour, while GelMA samples were crosslinked under UV light for 3 min (Supplementary Figure S5). To assess GelMA polymerization during photopolymerization, varying concentrations (3%–7%) were applied to 1/4EPI silicone substrates with different thinner ratios, and absorbance at 370 nm was measured before and after UV crosslinking. Results (Supplementary Figure S6) showed that both GelMA concentration and substrate softness significantly influenced light absorption and gel retention, highlighting the importance of mechanical compatibility in hybrid soft constructs.

### 2.6 Biological characterization

Cellular responses to GelMA hydrogels were evaluated using Live/Dead staining (Invitrogen™ LIVE/DEAD™ Viability/Cytotoxicity Kit) and the CCK8 assay (Sigma-Aldrich). A Nikon Eclipse Ti2 microscope was used for imaging. ImageJ software was employed to quantify live and dead cells and calculate cell viability as a percentage. The CCK8 assay was used to assess the metabolic activity of encapsulated cells. Results were normalized to negative controls and expressed as optical density (OD) values.

## 3 Results

### 3.1 Physical properties of the synthetic model

**FIGURE 2:**
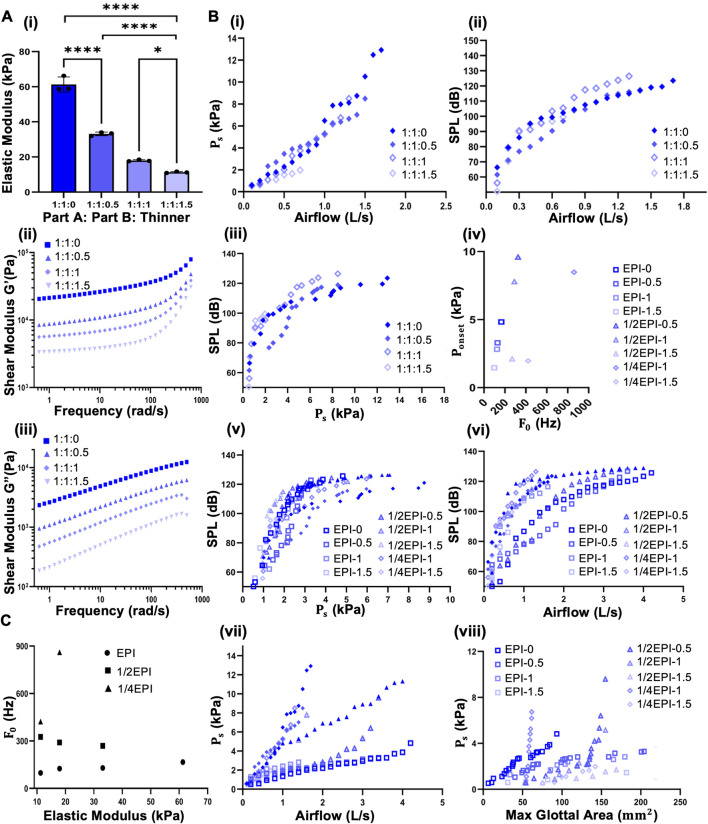
**(A)** Mechanical characterization of silicone rubber with varying silicone thinner ratios (1:1:0 to 1:1:1.5), showing elastic modulus (bar graph) and frequency-dependent storage (G′, i) and loss (G″, ii) moduli. One-way ANOVA confirms significant stiffness reduction with increasing thinner content. **(B)** Phonatory performance of the EPI 1:4 model: (i) subglottal pressure vs. airflow, (ii) sound pressure level (SPL) vs. airflow, (iii) SPL vs. subglottal pressure, and (iv) comparison of maximum glottal area across all EPI sizes and stiffnesses, indicating stiffness-dependent oscillatory behavior. **(C)** Correlation between fundamental frequency (F_0_) and material stiffness across models: (i) F_0_ vs. G′ and (ii) F_0_ vs. elastic modulus, showing that increased stiffness leads to higher phonatory frequencies.

Figure 2Ai–iii illustrates the effect of varying silicone thinner ratios (0, 0.5, 1.0, and 1.5 parts) on the mechanical properties of silicone rubber. As shown in Figure 2Ai, Elastic modulus decreases significantly with increasing thinner concentration. The modulus drops from approximately 65 kPa at a 1:1:0 ratio to below 20 kPa at a 1:1:1.5 ratio, indicating a substantial increase in material compliance and softness. Rheological characterization in Figure 2Aii,iii further supports this trend, with both the storage modulus (G′) and loss modulus (G″) decreasing consistently across the frequency spectrum (1–1,000 rad/s) as thinner content increases. These reductions in elastic and viscous components suggest a loss of structural rigidity and energy dissipation capacity. The formulations with ratios of 1:1:0.5 and 1:1:1 exhibit mechanical properties that align closely with the reported physiological range of human vocal fold tissue (10–40 kPa), depending on anatomical region and phonatory loading. This mechanical similarity makes them well-suited for use in bioengineered vocal fold replicas and dynamic bioreactor systems designed to emulate natural phonation mechanics. However, the viscoelastic parameters (G′, G″) were measured under small-strain conditions, in the linear viscoelastic regime. The self-sustained oscillations occur at high strain levels, which need to be considered when using the data for fluid-solid interaction modeling.

### 3.2 Phonatory characteristics of the synthetic model

The phonatory parameters are classified as subglottal pressure, which indicates the lung pressure (Hertegård et al., 1995; Sundberg et al., 2013), oscillation amplitude, and phonation range (Thomson, 2024; Thomson et al., 2005). Figure 2Bi–iii demonstrates that 1/4EPI replicas respond to material stiffness tuning, with softer silicone-thinner blends (1:1:1 and 1:1:1.5) reducing the phonation threshold but sacrificing maximum SPL performance. There was no oscillation in stiffer materials e.g., 0 and 0.5. These trade-offs are essential for tailoring bioengineered vocal fold replicas to mimic specific vibratory conditions relevant to human phonation. Supplementary Figure S3i illustrates the relationship between subglottal pressure (Ps) and airflow across different EPI model sizes—EPI, 1/2EPI, and 1/4EPI—each tested with varying silicone thinner ratios (0, 0.5, 1.0, and 1.5). As model size decreases, subglottal pressure increases for the same airflow, indicating greater resistance in smaller vocal folds. Among all groups, the 1/4EPI models exhibit the highest subglottal pressures, reaching up to ∼13 kPa at relatively low airflow (<2 L/s). As expected, EPI models show the lowest pressures. In the case of 1/2EPI, the pressure rises more steeply in the middle range of the airflow (>2 L/s) and is similar to the case of EPI for lower airflow values. The solid lines (e.g., 1/2EPI-0, 1/4EPI-0, and 1/4EPI-0.5) indicate configurations that did not produce self-sustained oscillation, reinforcing the need for adequate material compliance. Increasing the silicone thinner ratio within each model consistently reduces the subglottal pressure, due to increased compliance. The results highlight the role of viscoelastic properties in controlling the mechanical energy required for oscillations.

In Figure 2Biv–viii, the same mechanical-acoustic analysis was extended to all EPI sizes, including full-scale, 1/2, and 1/4 replicas, with all silicone ratios. The maximum glottal area expanded with increasing airflow (Figure 2Biv), but this response was more pronounced in miniaturized and more compliant replicas, reinforcing their lower resistance to deformation. Figure 2Bv shows that the sound pressure level (SPL) increases with airflow exponentially and plateaus at higher flow rates across all models. Interestingly, the 1/2EPI case consistently generated the highest SPL values, peaking above 130 dB, indicating optimal acoustic output. The EPI case produced lower SPLs, suggesting a limited vibratory capacity due to the volume of the glottal site. Higher silicone thinner ratios also lead to slightly lower SPLs, reflecting a trade-off between material softness and vibratory efficiency. The models that failed to oscillate under airflow (i.e., solid points) reflected passive airflow noise. In human vocal folds, the subglottal pressure typically ranges from approximately 5–10 cm H_2_O (0.5–1.0 kPa) during conversational speech, increasing to >20 cm H_2_O (>2.0 kPa) for loud voices (Döllinger et al., 2016). The 1/4EPI case reached subglottal pressures up to ∼13 kPa at airflow rates below 2 L/s, significantly exceeding physiological levels. The human voices usually produce 60–80 dB during normal speech and up to 100–110 dB during shouting or singing (Lee and Kim, 2019). The 1/2EPI models, however, exceed 130 dB, indicating enhanced acoustic output beyond natural ranges. These results suggest that while EPI models capture core vibratory dynamics, the length scale significantly regulates phonation conditions. Supplementary Figure S3i–vi further supports these findings with detailed stiffness-dependent trends. Supplementary Figure S3i–iii compares phonation behavior across silicone blends (1:1:0 to 1:1:1.5), showing increasing SPL and Ps with airflow and confirming that 1:1:1 and 1:1:1.5 yield oscillatory behavior. Supplementary Figure S3iv–vi specifically highlights the 1/2EPI size model, emphasizing that softening the replica reduces phonation threshold (lower Ps at low airflow), but compromises SPL at higher flows. These supplementary plots reinforce the need to balance stiffness and compliance when designing voice prostheses or vocal fold models, as excessively soft materials improve onset but reduce sustained sound output.

Image-based segmentation and area quantification were conducted to extract the subglottal area over time (FASTCAM Mini videos). The video was first selected by the user and read using VideoReader, with a frame rate of 20,000 frames per second. Each frame was converted to grayscale, and adaptive thresholding was applied to isolate the dark glottal region from the brighter surrounding replicas. The code calculated the number of pixels representing the glottal area. It converted it to a physical area in mm^2^ using a known pixel size (0.01 mm/pixel, yielding an area of 0.0001 mm^2^ per pixel). This conversion enabled tracking the dynamic glottal area frame-by-frame over a 0.02-s window. A moving average filter was then applied to smooth the extracted area data, reducing noise and improving interpretability. Figure 2Bviii shows that the maximum glottal area generally increased across all models as airflow increases, indicating greater vocal fold opening with stronger airflow. The 1/4EPI case (blue) and 1/4EPI case exhibit a steeper increase in glottal area at lower airflow levels compared to EPI, suggesting enhanced dynamic responsiveness in smaller or softer constructs. The compliant replicas or silicone thinner ratios regulate maximum areas earlier, implying that more areas can reduce the resistance to phonation and oscillations (Döllinger et al., 2016; Luizard et al., 2023).

Figure 2C illustrates that the fundamental frequency (F0) remains relatively low and stable (∼100–150 Hz) across a wide stiffness range, proposing limited frequency sensitivity to material properties. Compared to the typical human range of 125 Hz in adult males 70 and 210 Hz in adult females 72 under normal speech conditions, it almost falls between this range. Conversely, the 1/2EPI and 1/4EPI models show an evident increase in F0 as stiffness increases, with 1/4EPI models reaching over 1,200 Hz at ∼18 kPa, indicating a strong dependence on geometry and elasticity. The elevated frequencies in smaller, stiffer replicas resemble the pitch range observed in younger individuals and females (Sonninen and Hurme, 1998; Andersen et al., 2023; Zhang et al., 2007). The onset of oscillation, glottal area variation, and subglottal pressure–frequency relationship (Figures 2B,C) capture the material’s large-strain response under airflow excitation. The amplitude of fold opening during phonation corresponds to estimated surface strains above 15%, confirming that the system experiences large deformations.

Frequency tuning was achieved by altering the viscoelastic properties of silicone-based vocal fold replicas. The elastic modulus of the material was adjusted by varying the ratio of silicone thinner added to the base polymer mixture. Each ratio yielded a distinct stiffness profile, directly influencing the natural vibration frequency under identical airflow conditions. Frequency modulation was achieved passively through material stiffness tuning, without active mechanical actuation. By varying the silicone thinner ratio, the replica’s elastic modulus ranged from ∼65 kPa to ∼20 kPa, resulting in corresponding frequency shifts from ∼150 Hz to >1,200 Hz. This mechanical tuning approach enables reproducible, quantifiable control over vibratory response within a single geometric design.

### 3.3 Phonatory characteristics of the gelatin-synthetic composite

In the second phase of the experiment, the 1/4EPI case was used to investigate the influence of encapsulated hydrogel stiffness, injection volume, and base material compliance on phonatory behavior. Two groups were tested using different silicone thinner ratios—1% and 1.5% —while the encapsulated materials included Gelatin and GelMA at 3%, 5%, and 7% concentrations, with both 10 (Low) and 20 (High) volumes. A naming convention was adopted for clarity, where, for example, L3GN denotes Low 3% Gelatin and H5GM indicates High 5% GelMA.

**FIGURE 3:**
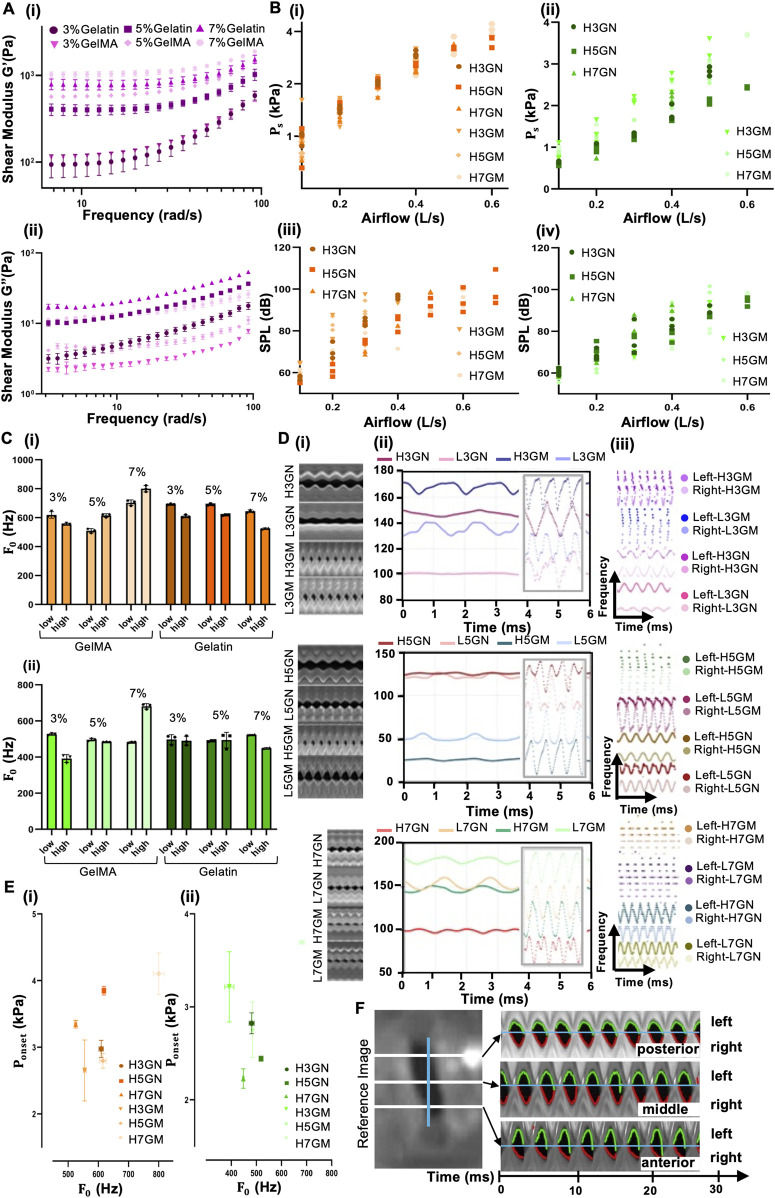
**(A)** Rheological properties of gelatin and GelMA hydrogels at 3%, 5%, and 7% concentrations, showing storage modulus G′ (i) and loss modulus G″ (ii) across angular frequency. **(B)** Phonatory behavior of high-volume (20 μL) Gelatin and GelMA vocal fold constructs under two silicone thinner ratios: orange (1%) and green (1.5%). Subglottal pressure vs. airflow for Gelatin (i) and GelMA (ii); sound pressure level (SPL) vs. airflow for Gelatin (iii) and GelMA (iv). **(C)** Fundamental frequency (F_0_) across material types and concentrations, comparing Gelatin and GelMA under 1% (i, orange) and 1.5% (ii, green) silicone ratios. **(D)** Representative kymograms (VKG), glottal opening waveform (GOFW), and vocal fold trajectories (VFT) for all material combinations and conditions. **(E)** Onset pressure (P_onset) plotted against F_0_ under 1% (i, orange) and 1.5% (ii, green) silicone thinner conditions. **(F)** Synchronized kymographic time series extracted from vocal fold oscillations showing symmetry and phase timing across conditions.

Gelatin and GelMA exhibit increasing stiffness with higher concentrations, as expected (Figure 3Ai). GelMA samples consistently show higher G′ values than their gelatin counterparts at equivalent concentrations, indicating a more elastic and crosslinked network structure. The case of 7% GelMA displays the highest storage modulus (>1,000 Pa), reflecting superior energy storage and structural rigidity, followed by 7% Gelatin. This trend is preserved across the frequency range, showing frequency-dependent viscoelastic reinforcement. Gelatin samples exhibited higher loss modulus (G″) at higher concentrations, particularly the 7% Gelatin, suggesting more energy loss as heat during deformation (Figure 3Aii). The GelMA case showed slightly lower G″ values due to the photocrosslinked network, which indicates more elastic-dominant behavior at all concentrations. These results confirm that increasing concentration enhances elastic and viscous properties. GelMA hydrogels are generally stiffer and more elastic than Gelatin at matched concentrations, consistent with their crosslinked network characteristics.

For clear data reporting Figure 3B is specified for higher volumes of different concentrations in which orange colors indicate 1% silicone, thinner and green color is an indication of 1.5% silicone thinner. In the subglottal pressure graphs Figure 3Bi,ii and Supplementary Figure 4Si,ii, a consistent increase was observed with rising airflow. The formulations with higher stiffness and volume (e.g., H7GN, H7GM) produced significantly higher pressures, suggesting greater flow resistance due to increased rigidity. In contrast, lower concentration and lower volume samples (e.g., L3GN, L3GM) exhibited more gradual pressure increases, indicating easier deformation and airflow accommodation. This trend was still apparent in the 1.5% thinner group, although the absolute subglottal pressure values were reduced due to the increased compliance of the surrounding silicone rubber material. The softer matrix allowed more expansion, thereby requiring less pressure to initiate vibration.

The SPL graphs [Figure 3Biii,iv and Supplementary Figure 4Siii,iv] reflected material and structural differences. Under the 1% silicone thinner condition (bottom left), high-stiffness, high-volume samples like H7GM and H5GM reached SPLs close to 120 dB, confirming their ability to sustain efficient oscillations and sound generation. Meanwhile, softer and smaller volume gels, particularly L3GN and L3GM, demonstrated noticeably lower SPLs across airflow levels, due to insufficient vibratory force or incomplete glottal closure. When the thinner ratio was increased to 1.5% (bottom right), SPLs generally decreased, especially for stiffer gels. This can be due to the more compliant base matrix absorbing vibratory energy and reducing coupling efficiency between the injected material and replica motion. Thus, higher stiffness and volume enhance phonatory power, while greater compliance from added silicone thinner lowers mechanical resistance but may reduce vibratory energy transfer. This tunable interaction enables tailored vocal fold replica design for diverse phonatory conditions.

In this experiment, the parameter F0 in the 1/4EPI case was evaluated under two base compliances—1% and 1.5% silicone thinner ratios—while varying encapsulated hydrogels’ type, concentration, and volume. Figure 3Ci, which corresponds to the 1% silicone thinner condition (stiffer base), GelMA-based replicas consistently exhibit higher F0 compared to those with Gelatin at the same concentration and volume. The highest F0 (∼750 Hz) is observed in the high-volume 5% GelMA group. Across GelMA samples, increasing hydrogel volume enhances F0, suggesting that greater embedded mass and stiffness promote faster oscillatory cycles. Gelatin samples, by contrast, show relatively modest increases in F0, with values ranging from approximately 350–500 Hz. This indicates that Gelatin, being mechanically softer and less elastic, contributes less to vibratory frequency amplification.

Figure 3Cii represents data from the 1.5% silicone thinner group, where the replica body is more compliant. Here, an overall rise in F0 is observed, particularly for high-concentration GelMA formulations. The high-volume 7% GelMA sample reaches >900 Hz, highlighting the synergistic effect of high stiffness and soft external constraint. Interestingly, the influence of volume becomes more pronounced in this condition. For Gelatin-embedded models, F0 significantly increases with injection volume even when concentration is low, indicating that within a softer matrix, bulk contributes more to vibrational control than stiffness alone (Duflo et al., 2006; Shaw et al., 2012; Wan-Chiew et al., 2021). The matrix’s mechanical properties (modulated by silicone thinner ratio) interact with the embedded gel’s viscoelasticity, allowing precise tuning of F0. This suggests that composite tuning of core stiffness, volume, and base compliance is essential for replicating a broad range of human phonatory behaviors in synthetic vocal fold models.

The high-speed video data were (Figure 3D) analyzed using image processing techniques adapted from Andrade-Miranda et al. (Luizard et al., 2023) to extract VKG, GOFW, and VFT. Kymograms were generated by extracting a horizontal scanline at the mid-glottal plane across all video frames, as defined below in Andrade-Miranda et al. The VKG matrix visualizes temporal changes in the glottal opening, with variations in symmetry and closure patterns between formulations. Samples such as H3GN and H5GM exhibited smooth, sinusoidal vibratory patterns, indicative of stable, symmetric oscillations. In contrast, flatter patterns (e.g., L3GM) suggested reduced vibratory amplitude or stiffness-induced restrictions in medial displacement, revealing temporal glottal opening/closure patterns.

$$I_{DKG}\left(x,y\right)=\left[KG\left(t_{1}\right),KG\left(t_{2}\right),\ldots ,KG\left(t_{n}\right)\right]$$

Motion fields were computed using optical flow estimation across the segmented glottal region, providing velocity information over time as formulated below. The vector displacement field $\vec{w}\left(x_{ij},t_{k}\right)$ captured glottal wall divergence and convergence. GOFW plots revealed clear distinctions between samples; higher GelMA concentrations (e.g., L6GM) produced larger amplitude excursions, while Gelatin-rich formulations demonstrated more damped motion, indicating softer, more compliant behavior to characterize dynamic fold displacement and energy transmission.

$$\vec{w}\left(x_{ij},t_{k}\right)=\left(u\left(x_{ij},t_{k}\right),v\left(x_{ij},t_{k}\right)\right)$$

Using the vocal fold contour extraction approach described above, we computed left and right vocal fold deflection trajectories $\delta_{l,r}^{seg}\left(pc,t_{k}\right)$ at a constant anterior-posterior location (typically 50% along the glottal axis). The resulting VFT plots allowed quantification of cycle periodicity, amplitude, and interfold symmetry. Conditions such as L5GM and H7GN showed well-defined, phase-aligned left and right trajectories, whereas L3GM and L7GM exhibited reduced synchronization and asymmetry, due to increased viscoelastic heterogeneity.

$$\delta_{l,r}^{seg}\left(pc,t_{k}\right)={\left\|g_{pc}\left(t_{k}\right)-c_{l,r,pc}\left(t_{k}\right)\right\|}_{2}$$

These playback metrics not only supported the qualitative visual interpretation of oscillatory function but also enabled objective comparison across formulations. The use of these high-resolution representations, in alignment with established HSV-based vibratory analysis protocols, enhances the reliability of phonatory assessments in synthetic vocal fold models and supports the development of tunable materials for bio-inspired laryngeal prosthetics. Find all kymograms in Supplementary Figure S7.

Figure 3E presents a comparison between onset pressure (the subglottal pressure required to initiate vocal fold oscillation) and the resulting fundamental frequency (F0) for various hydrogel formulations embedded in 1/4EPI vocal fold replicas, under two different base material conditions: 1% silicone thinner (right panel. Figure 3Ei) and 1.5% silicone thinner (left panel. Figure 3Eii). Each data point represents the meaning of three repeated trials, with error bars showing variability in both pressure and frequency. The symbols and colors correspond to specific formulations defined by material type (Gelatin or GelMA), concentration (3%, 5%, 7%), and injected volume (Low: 10 (Supplementary Figure 5Si,ii), High: 20). In the 1% silicone thinner group (right panel), the onset pressures generally range from 2.0 to 3.8 kPa. A modest inverse trend can be observed where samples with higher F0 tend to require lower onset pressures, particularly for stiffer or higher-volume GelMA formulations (e.g., H7GM, H5GM). This may suggest more efficient energy transfer in these configurations. Gelatin-based replicas, such as L3GN and L5GN, cluster around lower F0 values (∼400–500 Hz) but exhibit more variability in onset pressure, reflecting their less predictable oscillation behavior due to lower stiffness.

In the 1.5% silicone thinner group (left panel), onset pressures span a slightly broader range (∼2.0–4.5 kPa), but with a clearer pattern: stiffer, higher-concentration gels like H7GN and H7GM consistently achieve higher frequencies (>700 Hz) while maintaining moderate onset pressures, demonstrating efficient phonation even in a more compliant matrix. Low-stiffness samples such as L3GN and L3GM show higher onset pressures with lower F0, indicating that softer materials require more subglottal pressure to initiate vibration and are less efficient acoustically. Additionally, some Gelatin groups exhibit higher onset variability, reflecting inconsistencies in vibratory behavior due to less stable material properties.

Figure 3F displays a spatiotemporal analysis of vocal fold motion captured from a high-speed video recording, illustrating glottal dynamics along three anatomical positions—posterior, middle, and anterior—over time. On the left, a reference image of the glottal area is shown with three horizontal lines indicating the exact regions from which kymograms were extracted. These kymograms provide time-series visualizations of glottal edge movements from the left and right vocal folds over a duration of approximately 50 milliseconds.

In the kymograms on the right, each frame shows periodic vocal fold oscillations as they open and close. The left and right fold edges are traced in green and red over the grayscale glottal space, revealing the symmetric and cyclic vibratory behavior. The waves are mostly sinusoidal, with consistent amplitude and phase across the anterior–posterior axis, indicating stable phonatory conditions. Small phase differences between the posterior and anterior segments may be observable, reflecting physiological wave propagation or minor asymmetries in fold stiffness or boundary constraints. This type of representation is highly valuable for assessing mucosal wave propagation, vibratory symmetry, and spatial variations in oscillatory timing, which are critical for understanding normal versus pathological phonation.

### 3.4 Biological response of the gelatin-synthetic composite

**FIGURE 4:**
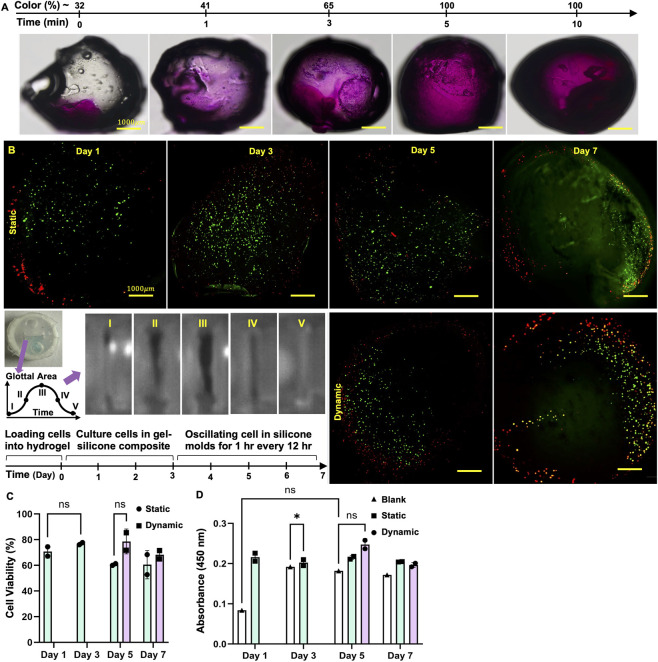
**(A)** Time-lapse diffusion of Rhodamine B in GelMA constructs under airflow-induced oscillation, showing progressive dye spread from ∼32% to ∼100% coverage over 10 min. **(B)** Confocal Live/Dead assay images (×4 magnification) showing cell viability in 5% GelMA constructs under static and vibratory conditions across Days 1, 3, 5, and 7; green: live cells, red: dead cells (scale bar is 200 μm). **(C)** Quantification of cell viability (%) based on Live/Dead imaging, with no significant differences between static and vibration groups over time. **(D)** CCK-8 absorbance (450 nm) indicating metabolic activity, showing significant increases in the vibration group at Days 5 and 7 compared to earlier time points. Data are presented as mean ± SD; *p < 0.05, **p < 0.01, ns: not significant.

To evaluate the mechanoresponsive behavior of GelMA and its ability to propagate vibratory cues through its matrix, Rhodamine B isothiocyanate was deposited asymmetrically on one side of a 5% GelMA construct. The sample was then subjected to controlled airflow-induced oscillation for varying durations (0, 1, 3, 5, and 10 min), and brightfield images were captured to visualize the dye distribution over time. The purple-stained region represents the diffusion of Rhodamine into the hydrogel network, indirectly visualizing the effect of mechanical motion on mass transport. At 0 min (Figure 4A), Rhodamine remains localized at the initial deposition site. After 1 min of oscillation, approximately 32% of the constructed areas show dye diffusion, which increases to ∼41% by 3 min. A sharp rise in distribution is observed at 5 min, with diffusion covering nearly the entire GelMA volume (∼100%), which remains unchanged through 10 min. This rapid increase between 3 and 5 min suggests that oscillatory motion significantly accelerates solute transport within the hydrogel. The findings support the hypothesis that GelMA can effectively sense and transmit mechanical vibrations, enabling dynamic redistribution of molecular signals—a property highly relevant for mimicking mechanically active tissue environments such as the vocal folds. For more information, the Z-stack is done with Confocal as shown in Supplementary Figure 6S.

After proving that the hydrogel inside the replicas is sensing the vibration from Airflow, 3T3 cells were encapsulated in 5% GelMA hydrogels (1 million cells per construct), with each construct containing 20 μL of hydrogel (high-volume condition). For the initial trial, cells are cultured at Day 1, and the sample oscillates for 2 hours a day on each of Day 4- Day 7. Supplementary Figure 7S demonstrates the cell viability (%) for each fold across all days, showing an initial high viability followed by a gradual decline due to cumulative mechanical stimulation. The samples were initially cultured under static conditions for Day 1- Day 3, followed by exposure to airflow-induced oscillation for 2 hours at Day 4. Live/Dead assays were performed at Day 5 using confocal microscopy to assess cell viability. The experiment was repeated at Day 6 with an additional 2-h vibration, and the final viability was evaluated at Day 7 (Latifi et al., 2016). Static controls were maintained for comparison at each time point, and each condition was tested in triplicate.

The confocal images (Figure 4B) show green fluorescence indicating live cells and red fluorescence indicating dead cells. Under static conditions, cell viability appears relatively consistent from Day 1 through Day 5, with an increase in live cell density by Day 3 and a modest decline by Day 7. In contrast, the vibrated samples at Day 5 and 7 show localized clusters of dead cells, particularly along the peripheral regions interfacing with silicone rubber. This suggests that mechanical stimulation alone does not compromise viability, but when combined with direct contact with the elastomer, cytotoxicity may arise due to leaching uncured components or material incompatibility.

By Day 7, the vibrated samples exhibit a more pronounced zone of cell death near the contact interface, reinforcing the hypothesis that silicone rubber may pose localized toxicity under prolonged culture or repeated mechanical loading. These observations highlight the importance of material biocompatibility at tissue–substrate junctions, particularly in oscillatory environments that mimic physiological vocal fold motion.

The Cell Counting Kit-8 (CCK-8) assay is a colorimetric method used to assess cell viability and proliferation by measuring metabolic activity. Viable cells reduce the water-soluble tetrazolium salt (WST-8) to an orange-colored formazan product, which can be quantified by absorbance at 450 nm. The more metabolically active the cells are, the greater the absorbance signal. In this study, absorbance from the Blank group (cell-free GelMA constructs) was used to control background signals from the hydrogel media components.

Figure 4C presents cell viability percentages over four time points (Days 1, 3, 5, and 7) for 3T3 fibroblasts encapsulated in 5% GelMA, comparing static and vibration conditions. Data were quantified using ImageJ from confocal Live/Dead assay images, with green fluorescence representing live cells and red indicating dead cells. At Day 1, the static condition shows slightly higher viability (∼74%) compared to the vibration group (∼69%), indicating that initial exposure to vibration may cause mild cellular stress or adaptation delay. By Day 3, both groups show elevated viability (∼82% static vs. ∼80% vibration), suggesting successful early adaptation and healthy proliferation under both conditions, and a divergence appears by Day 5, where the static group maintains high viability (∼85%). In comparison, the vibration group drops noticeably (∼61%), potentially indicating that prolonged or repeated mechanical stimulation may lead to mild cellular fatigue, apoptosis, or stress-related responses. By Day 7, the trend persists: the vibration group remains slightly lower (∼68%) compared to the static group (∼72%), though the difference is less pronounced. Overall, the data suggest that while short-term vibration does not significantly impair viability, repeated mechanical stimulation may gradually reduce cell viability, especially after Day 3. This indicates that while GelMA-embedded cells tolerate early vibratory cues well, optimization of oscillation duration, frequency, or rest periods may be required for long-term culture stability.

The CCK-8 assay results (Figure 4D) demonstrate distinct trends in cell metabolic activity across static, vibration, and blank (control) conditions. The blank group, consisting of acellular GelMA constructs, showed consistently low absorbance values over time, confirming minimal background interference from the hydrogel matrix and validating that the measured signals in the experimental groups primarily reflect cellular activity. In the static group, absorbance remained relatively stable from Day 1 through Day 7, indicating sustained cell viability without significant proliferation. In contrast, the vibration group exhibited a marked increase in absorbance by Day 5, suggesting that mechanical stimulation enhanced cellular metabolism or proliferation, likely through mechanotransductive pathways. Although a slight decline was observed by Day 7, the absorbance remained higher than in the static group, indicating that cells remained metabolically active. These results support the conclusion that short-term oscillatory stimulation promotes cell function within GelMA hydrogels, while the blank controls confirm the specificity of the observed response to viable cells.

## 4 Conclusion

This study successfully demonstrates the development and validation of a vocal fold replica system composed of silicone rubber and GelMA hydrogels, designed to investigate the impact of oscillatory airflow on hydrogel behavior and cell viability. Through a series of controlled experiments, we confirmed that mechanical stimulation via airflow can induce measurable solute diffusion, hydrogel deformation, and cellular responses within the 3D constructs. Rhodamine B diffusion under oscillatory stimulation provided visual and quantitative evidence of mechanoresponsive transport within GelMA, reinforcing its suitability as a biologically relevant material for mimicking vocal fold-like environments.

The EPI geometry represents a simplified convergent–divergent model of the glottis during phonation. This configuration is sufficient to reproduce the fluid–structure interactions that drive self-sustained oscillations. By abstracting the complex anatomical features of the human larynx, the model isolates the primary physical mechanisms underlying phonatory behavior—namely, the coupling between subglottal airflow, pressure asymmetry, and tissue deformation. The system thus serves as a controllable and biomimetic analog that prioritizes mechanical tunability and reproducibility over anatomical fidelity.

We further explored the biological implications of this system by encapsulating 3T3 fibroblasts within 5% GelMA and subjecting them to periodic vibration over a multi-day culture period. Live/Dead assays and CCK-8 analyses revealed that moderate, short-term oscillation could enhance metabolic activity, as seen by elevated absorbance levels by Day 5. However, extended or repeated vibration led to localized reductions in cell viability, particularly near the rubber interface, likely due to material-related cytotoxicity or mechanical fatigue. These findings underscore the importance of tuning mechanical properties and oscillation protocols to balance cellular health and mechanotransduction.

Our results demonstrate that the platform can be adapted to encapsulate biological cells for extended experimental studies. Although the enclosed microenvironment surrounding the spherical region limits the efficient exchange of oxygen and other nutrients, periodic replenishment of the culture medium proved an effective strategy for maintaining cellular viability and overall platform functionality. The follow-up work will focus on investigating microstructural variations under physiologically relevant conditions, thereby enabling more profound exploration of the platform’s biophysical and biological interactions.

In conclusion, this platform establishes a foundation for investigating biomechanical stimulation in vocal fold tissue engineering and offers valuable guidance for designing hydrogel-based systems with controlled oscillation. There are some limitations with this work. One limitation of our platform is the lack of tissue inhomogeneity and anisotropy. Regarding tissue inhomogeneity, this platform is a two-material system with limited control over its physical properties, while the tissue can be divided into five areas: epithelium, superficial lamina propria, intermediate lamina propria, deep lamina propria, and vocalis muscle. In terms of anisotropy, our material system does not reflect the orientation of the muscular network. Future work will involve adding fibers to the system to induce both tissue anisotropy and the muscular compartment. This can be done via synthesizing fibers and mixing with our rubber system. Other studies may focus on incorporating human vocal fold epithelial cells, fine-tuning the mechanical properties of the elastomer-hydrogel interface, and applying gene expression assays to capture long-term mechanobiological responses. The findings highlight GelMA’s potential as a mechanoresponsive scaffold for simulating the vocal fold microenvironment and offer a promising step toward preclinical voice tissue models.

## Data Availability

The raw data supporting the conclusions of this article will be made available by the authors, without undue reservation.

## Supplementary material

SUPPLEMENTARY FIGURE S1 Fabrication of a Miniaturized Vocal Fold Replica — The process begins with designing the mold in CAD and slicing it using software compatible with the 3D printer. The mold is then printed using a thermally resistant material (PLA in this case). Next, the polymer mixture is prepared by combining EcoFlex parts A and B with silicone thinner in varying ratios (0, 0.5, 1.0, 1.5). The materials are mixed thoroughly until small white bubbles appear. The mixture is then poured into the printed mold, along with spherical purls and a ring; applying oil to the mold beforehand aids in easy demolding. The mold is left for 1 hour to allow air bubbles to rise and escape, then polymerized in an oven for 3 min. After curing, the mold is partially cut to extract the purls, and the cut section is resealed using the same material. The final replica is ready for use and can be injected with different materials as needed.

SUPPLEMENTARY FIGURE S2 Fabrication of EPI and 50% EPI Vocal Fold Replica — The fabrication process is same. The only difference is we did not consider composite material for these sizes of structures as our aims were focusing on the miniaturized design.

SUPPLEMENTARY FIGURE S3 (i–iii) Phonatory performance of all EPI sizes under varying silicone thinner ratios, showing SPL vs. Ps (i), SPL vs. airflow (ii), and Ps vs. airflow (iii). (iv–vi) Detailed plots for EPI 1:2: Ps vs. airflow (iv), SPL vs. airflow (v), and SPL vs. Ps (vi). (vii–ix) Data for full-sized EPI: Ps vs. airflow (vii), SPL vs. airflow (viii), and SPL vs. Ps (ix), highlighting stiffness-dependent phonation thresholds and acoustic output.

SUPPLEMENTARY FIGURE S4 Phonatory characterization of low-volume GelMA and Gelatin constructs under two silicone thinner conditions. Orange markers represent 1% silicone thinner; green markers represent 1.5% thinner. (i–ii) Subglottal pressure vs. airflow for Gelatin (i) and GelMA (ii). (iii–iv) SPL vs. airflow for Gelatin (iii) and GelMA (iv). (v–vi) Phonation onset pressure as a function of F0 for Gelatin (v) and GelMA (vi), showing stiffness- and thinner-dependent differences in phonation efficiency.

SUPPLEMENTARY FIGURE S5 Effect of UV crosslinking time on the mechanical and phonatory properties of 5% GelMA hydrogels. Left and middle: Storage modulus (G′) as a function of angular frequency for 1, 2, and 3 min of UV exposure, showing increased stiffness with longer exposure time. Right: Corresponding changes in fundamental frequency (F0) and phonation onset pressure (P_onset) over time, highlighting the influence of crosslinking on vibratory performance.(The graph illustrates the effect of UV crosslinking time on the mechanical and phonatory properties of 5% GelMA hydrogels. Rheological measurements show that the storage modulus (G′) increases with longer UV exposure (1 min <2 min <3 min), indicating progressive stiffening due to enhanced crosslinking density. However, the phonatory response does not follow a linear trend: while the fundamental frequency (F0) initially increases, it declines after prolonged exposure, and the phonation onset pressure (P_onset) peaks at 2 min before decreasing. This suggests that excessive stiffness may hinder effective vibration, reducing F0 and requiring lower onset pressure due to limited deformation. Overall, the data highlight a mechanical–acoustic trade-off, indicating that moderate UV exposure (∼2 min) yields an optimal balance between structural integrity and phonatory efficiency for 5% GelMA vocal fold constructs).

SUPPLEMENTARY FIGURE S6 To evaluate the diffusion and retention behavior of GelMA within soft silicone substrates during photopolymerization, we encapsulated 3%, 5%, and 7% GelMA hydrogels on top of pre-cured 25% EPI structures with varying silicone thinner ratios (1:1:0, 1:1:0.5, 1:1:1, and 1:1:1.5). The experiments were conducted using a 96-well plate format, where each well was filled with a specific Ecoflex formulation, and a droplet of GelMA solution was deposited on the surface. Prior to crosslinking, absorbance at 370 nm was measured using an Agilent spectrophotometer to quantify the baseline optical density and assess initial GelMA-silicone interactions. Following this, the samples were exposed to UV light for 2 min to initiate crosslinking of the GelMA. Post-crosslinking absorbance was again measured at 370 nm to monitor changes in light absorption, potentially caused by gel solidification, changes in diffusion, or photochemical stabilization at the silicone interface. The “Before Crosslinking” data show that absorbance slightly increases with higher GelMA concentrations and greater silicone thinner content, likely due to increased gel penetration and light scattering at the interface. In contrast, the “After Crosslinking” data reveal a more pronounced difference: absorbance values generally decrease with higher thinner content, especially for lower GelMA concentrations, suggesting either enhanced diffusion into softer silicone matrices or reduced surface retention after UV curing. These results indicate that both GelMA concentration and substrate stiffness (tuned by silicone thinner ratio) significantly influence the optical density and crosslinking behavior of the encapsulated hydrogel. The findings underscore the importance of tuning the mechanical compatibility between hydrogel and substrate when designing hybrid soft tissue constructs.

SUPPLEMENTARY FIGURE S7 The kymograms shown represent the oscillatory behavior of vocal fold replicas fabricated with varying combinations of GelMA and Gelatin concentrations (3%, 5%, and 7%), silicone thinner ratios (1.0% and 1.5%), and capsule volumes (Low and High). Overall, conditions using 1.0% silicone thinner exhibited more prominent and well-defined sinusoidal waveforms compared to those with 1.5% thinner, suggesting that increased base stiffness (lower thinner content) enhances vibratory performance by providing sufficient resistance to support fold motion. In contrast, kymograms from 1.5% thinner conditions appear more damped and shallow, indicating that excessive compliance in the substrate hinders effective vibration. The capsule volume also played a significant role: High-volume encapsulation generally resulted in wider oscillation bands with greater contrast, reflecting stronger and more coherent fold motion. In contrast, Low-volume conditions often showed flatter or less periodic patterns, likely due to reduced hydrogel mass and tension, which diminishes the ability of the fold to sustain self-oscillation under airflow. When comparing Gelatin vs. GelMA, both materials performed best at 5% concentration under High-1.0% thinner conditions, with 5% GelMA in particular showing strong, symmetric vibration patterns. Conversely, 7% concentrations, particularly in GelMA, led to diminished oscillation across most conditions, suggesting that over-stiffening restricts dynamic displacement. Altogether, the clearest and most functional vibratory behavior was observed in combinations of moderate stiffness (5%), higher capsule volume, and stiffer base layers (1.0% thinner), highlighting the importance of mechanical matching between the hydrogel and elastomer for optimal phonation in vocal fold replicas.

SUPPLEMENTARY FIGURE S8 Z-stack confocal imaging of Rhodamine diffusion in GelMA hydrogel under airflow-induced oscillation at varying time points. To demonstrate that GelMA responds to oscillatory motion, a single Rhodamine droplet was introduced at a random location within a GelMA construct and subjected to airflow-induced vibration. Confocal Z-stack images were acquired at 0, 1, 3, 5, and 10 min using a laser-scanning confocal microscope. The progressive dispersion of Rhodamine fluorescence indicates that oscillatory forces facilitate molecular transport within the hydrogel matrix.

SUPPLEMENTARY FIGURE S9 Live/Dead confocal imaging of 3T3 fibroblasts encapsulated in 5% GelMA hydrogel under static and vibratory conditions. 3T3 cells were embedded in 5% GelMA and cultured under static conditions from Day 1 to Day 7. Vibration was applied for 2 h on Day 4 and again on Days 5 and 6. Live/Dead assays were performed using confocal microscopy (×10 objective) on Days 1, 3, 5, and 7 to evaluate cell viability. Green fluorescence indicates live cells, while red fluorescence marks dead cells. Static conditions show a gradual reduction in viability over time, whereas samples exposed to vibration on Days 4–6 were assessed post-exposure on Days 5 and 7, revealing differences in cellular response to mechanical stimulation.

SUPPLEMENTARY FIGURE S10 Live/Dead confocal imaging and quantitative analysis of cell viability in oscillated 3T3-encapsulated vocal fold replicas over 7 days. Vocal fold-shaped constructs encapsulating 3T3 fibroblasts within 5% GelMA were subjected to daily airflow-induced oscillation. Live/Dead assays were performed each day using confocal microscopy at both ×4 and ×10 magnifications to assess spatial viability in the left and right vocal folds (VF). Green fluorescence indicates viable cells, while red fluorescence indicates dead cells. The graph on the right quantifies cell viability (%) for each fold across all days, showing an initial high viability followed by a gradual decline due to cumulative mechanical stimulation. Right and left folds exhibited slightly different viability profiles, highlighting spatial variation in cellular response to oscillatory stress. Scale bars = 1,000 μm (4×) and 250 μm (10×).

SUPPLEMENTARY TABLE S1 Summary of oscillation presence across different EPI model sizes (EPI, 50%EPI, and 25% EPI) and silicone thinner ratios (1:1:0, 1:1:0.5, 1:1:1, 1:1:1.5). Cell with the F0 value indicate successful self-sustained oscillation under airflow, while N/A indicate the absence of oscillation. Results show that higher silicone thinner content generally promotes vibratory behavior, particularly in smaller models.
