# Supplementary material for: A hybrid vocal fold phonatory platform for pediatric phonation modeling
Source: Front Bioeng Biotechnol. 2026 Jan 21;13:1699406. doi: 10.3389/fbioe.2025.1699406 (PMC12895111; doi:10.3389/fbioe.2025.1699406)
Supplement: Supplementary file 1 [file DataSheet1.pdf]

SUPPLEMENTARY DATA

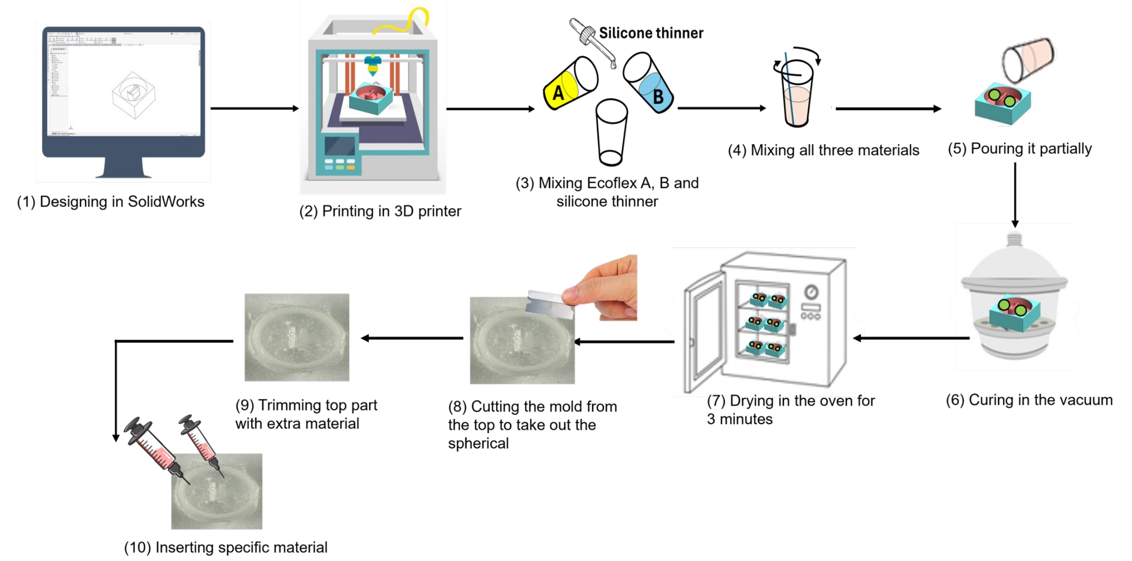

Figure S1

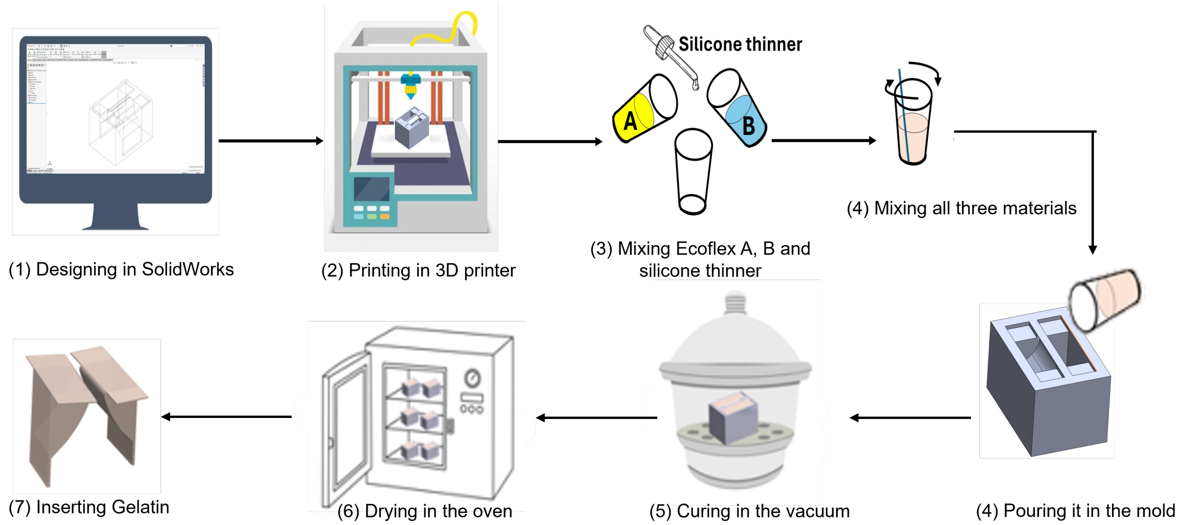

Figure S2

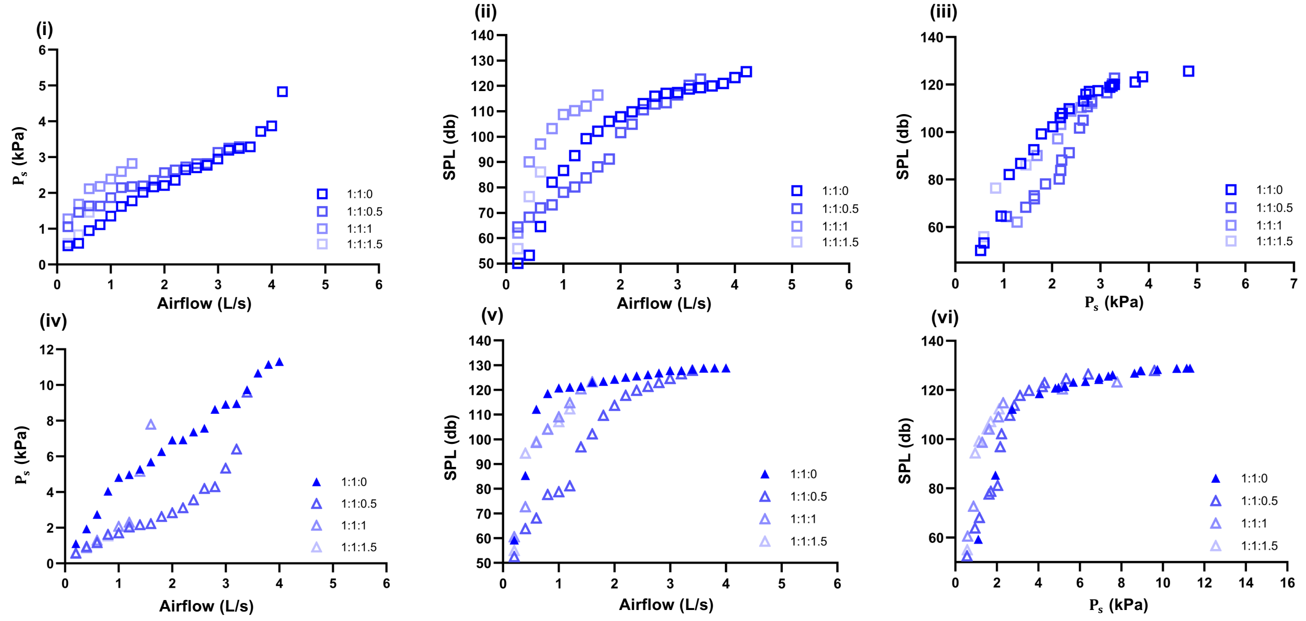

Figure S3

	Onset Fundamental Frequency (Hz)		
	EPI	1/2EPI	1/4EPI
Part A: Part B: Thinner			
1:1:0	171	N/A	N/A
1:1:0.5	130	325	N/A
1:1:1	122	290	860
1:1:1.5	97	268	422

Table S1

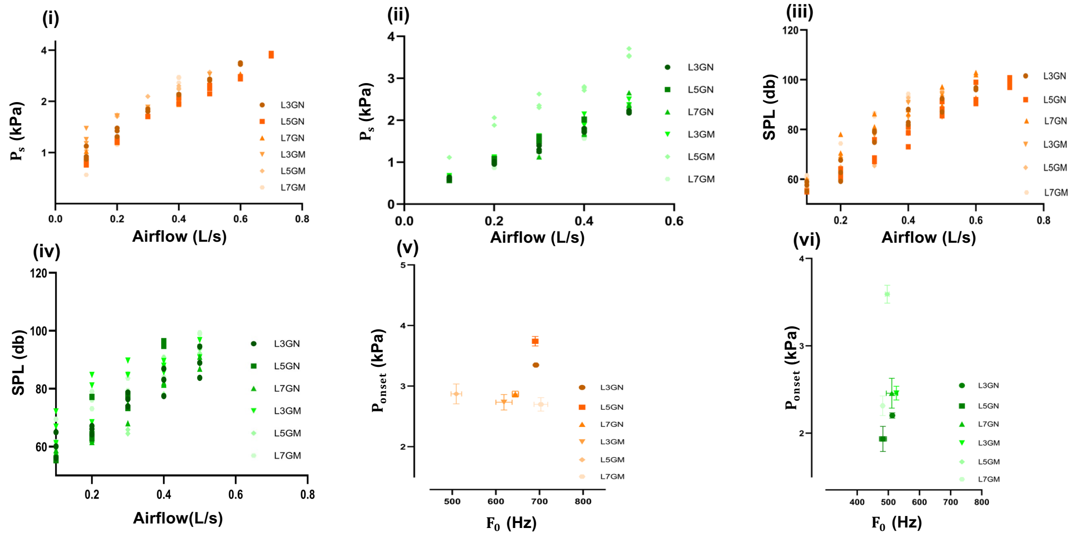

Figure S4

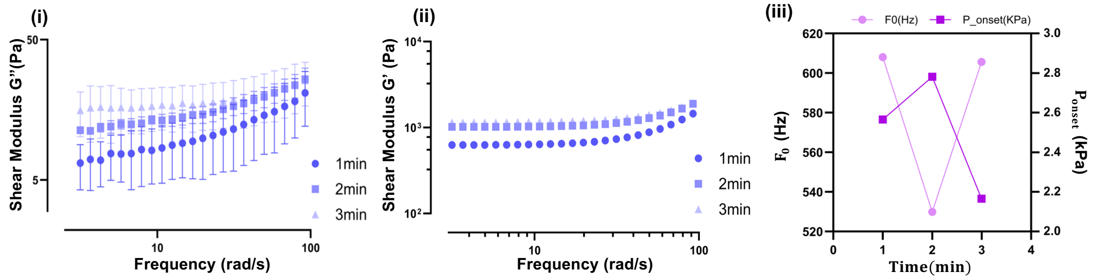

Figure S5

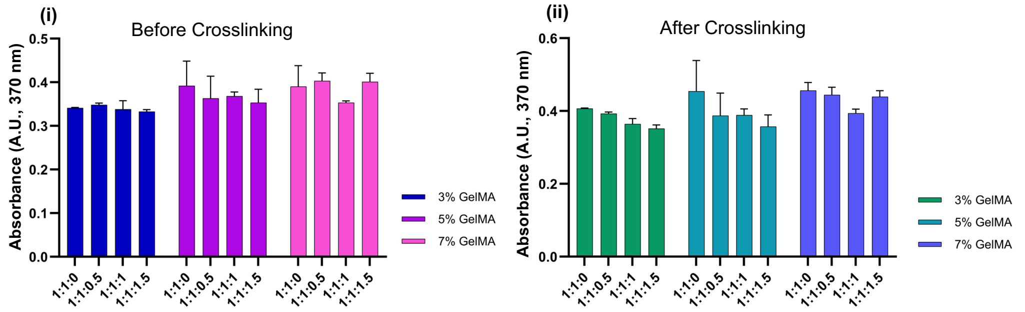

Figure S6

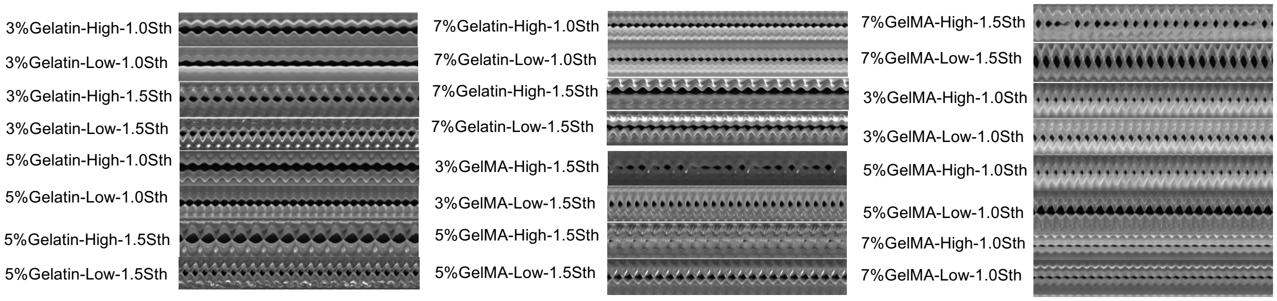

Figure S7

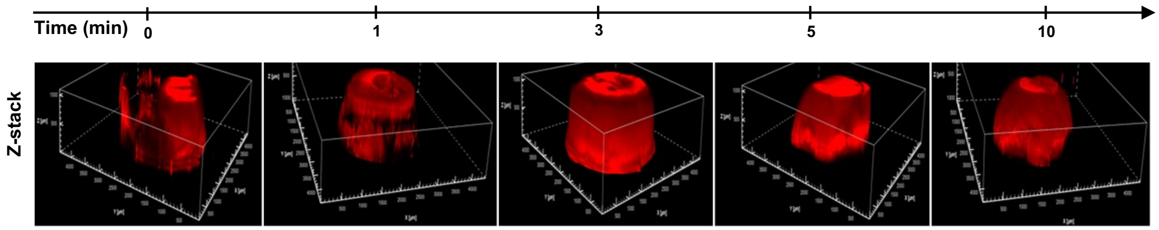

Figure S8

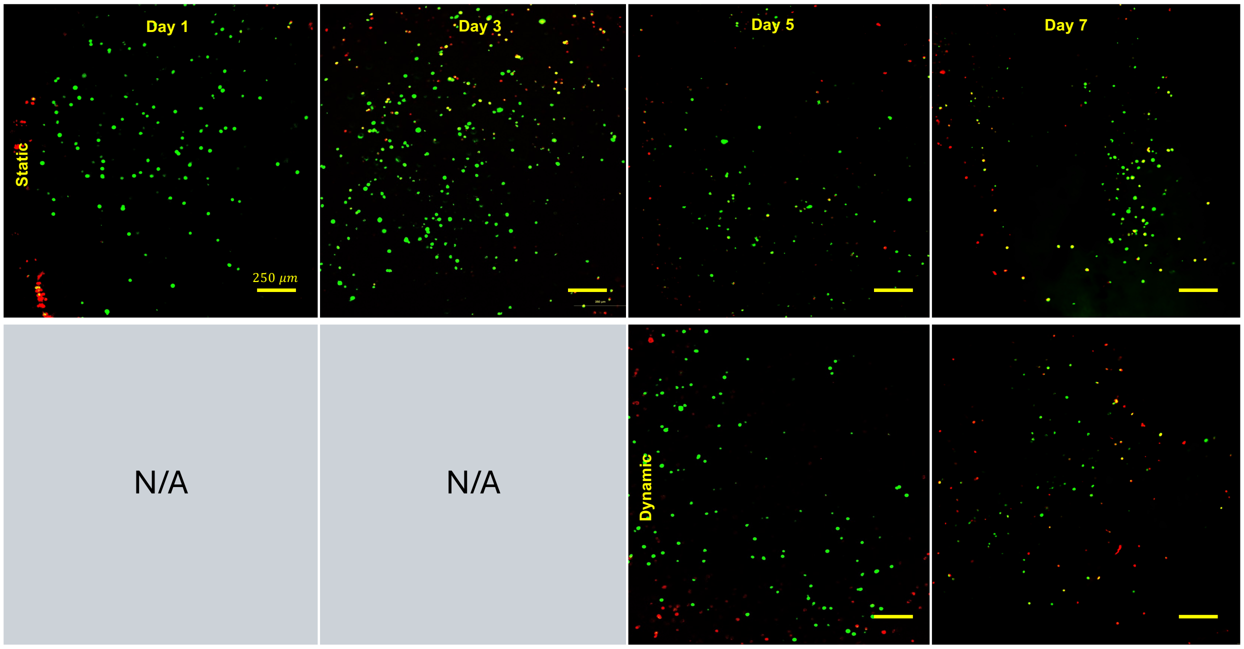

Figure S9

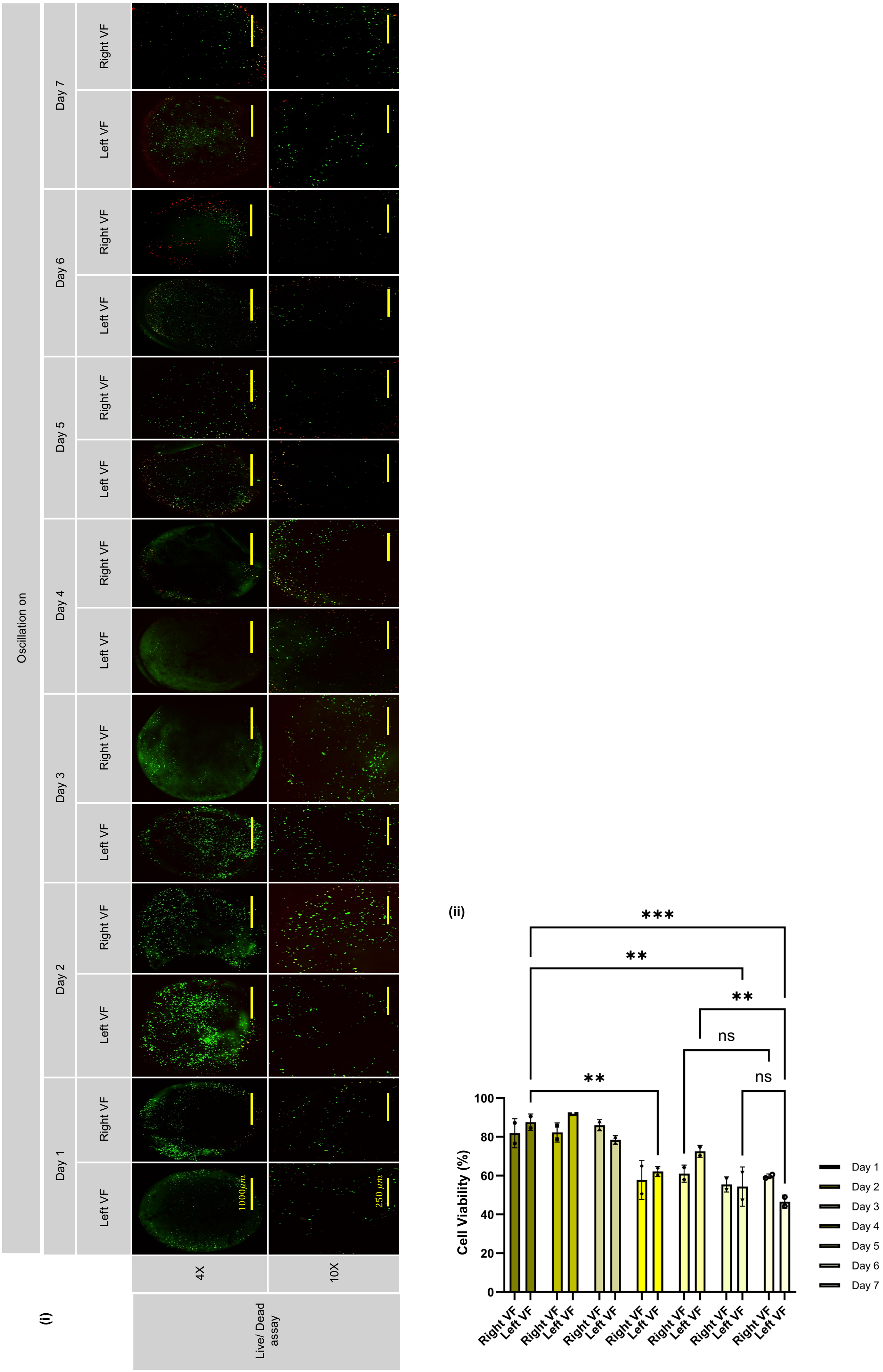
(i)
Oscillation on
Day 1
Day 2
Day 3
Day 4
Day 5
Day 6
Day 7
Left VF
Right VF
1000μm
250 μm
4X
10X
Live/ Dead assay
(ii)
***
**
**
ns
**
ns
Cell Viability (%)
0
20
40
60
80
100
Right VF
Left VF
Day 1
Day 2
Day 3
Day 4
Day 5
Day 6
Day 7

Figure S10

Figure S1. Fabrication of a miniaturized vocal fold (1/4EPI) replica — The process begins with designing the mold in CAD and slicing it using software compatible with the 3D printer. The mold is then printed using a thermally resistant material. The polymer mixture is then prepared by combining EcoFlex Part A and Part B (1:1 ratio) with four silicone thinner ratios: 0, 0.5, 1.0, and 1.5. The materials are mixed thoroughly until small white bubbles appear. The mixture is then poured into the printed mold, along with spherical purls and a ring. Oil is applied to the mold beforehand to ease demolding. The mold is left for one hour to allow air bubbles to rise and escape, then polymerized in an oven for three minutes. After curing, the mold is partially cut to extract the purls, and the cut section is resealed using the same material. The replica is ready for use and can be injected with different materials as needed.

Figure S2. Fabrication of EPI and 1/2EPI vocal fold replica — The fabrication process is similar to that in Figure S1. We did not consider composite materials for these structural sizes because we focused on the miniaturized design.

Figure S3. (i–iii) Phonatory performance of all EPI cases under varying silicone ratios, showing SPL vs. subglottal pressure (P_s) (i), SPL vs. airflow (ii), and P_s vs. airflow (iii). (iv–vi) Detailed plots for 1/2EPI P_s vs. airflow (iv), SPL vs. airflow (v), and SPL vs. P_s (vi).

Table S1. Summary of dynamic conditioning on fundamental frequency of phonation: Three EPI model sizes (EPI, 1/2EPI, and 1/4EPI) and four silicone Part A: Part B: Thinner ratios (1:1:0, 1:1:0.5, 1:1:1, and 1:1:1.5) were compared. The N/A suggests the absence of any phonatory oscillations.

Figure S4. Phonatory characterization of low-volume GelMA and Gelatin constructs under two thinner concentrations: Orange markers represent a 1:1:1 ratio; green markers represent a 1:1:1.5 ratio. (i–ii) Subglottal pressure (P_s) vs. airflow for Gelatin (i) and GelMA (ii). (iii–iv) SPL vs. airflow for Gelatin (iii) and GelMA (iv). (v–vi) Phonation onset pressure (P_{onset}) as a function of F_0 for Gelatin (v) and GelMA (vi), showing stiffness- and thinner-dependent differences in phonation efficiency.

Figure S5. Effect of UV crosslinking time on hydrogel performance: (i) loss modulus (G″) and (ii) storage modulus (G′) for 5% w/v GelMA as a function of angular frequency for 1, 2, and 3 minutes of UV exposure. (iii) Corresponding changes in fundamental frequency (F_0) and phonation onset pressure (P_{onset}) over time.

Figure S6. Absorbance (A.U. means arbitrary unit) of 3%, 5%, and 7% w/v GelMA hydrogels within soft silicone substrates for pre-cured 1/4EPI structures with four silicone Part A: Part B: Thinner ratios: 1:1:0, 1:1:0.5, 1:1:1, and 1:1:1.5. (i) Prior to crosslinking, absorbance at 370 nm was measured using an Agilent spectrophotometer to quantify the baseline optical density and assess initial hydrogel-silicone interactions. The samples were exposed to UV light for 2 minutes to initiate crosslinking of the hydrogel. (ii) Post-crosslinking absorbance was again measured at 370 nm to monitor changes in light absorption, potentially caused by gel solidification, changes in diffusion, or photochemical stabilization at the silicone interface.

Figure S7. The kymograms shown represent the oscillatory behavior of vocal fold replicas fabricated with varying combinations of GelMA and Gelatin concentrations (3%, 5%, and 7% w/v), silicone ingredient ratios (1:1:1 and 1:1:1.5), and capsule volumes (Low and High).

Figure S8. 3D perspective images of Rhodamine diffusion in GelMA hydrogel under airflow-induced oscillation at varying time points. Confocal Z-stack images were acquired at 0, 1, 3, 5, and 10 minutes.

Figure S9. Live/Dead confocal imaging of 3T3 fibroblasts encapsulated in 5% GelMA hydrogel under static and dynamic conditions. 3T3 cells were embedded in 5% GelMA and cultured under static conditions from Day 1 to Day 7. Dynamic conditioning was applied for 2 hours on Day 4 and again on Days 5 and 6. N/A means no dynamic conditioning happened.

Figure S10. (i) Live/Dead confocal imaging and quantitative analysis of cell viability in oscillated 3T3-encapsulated vocal fold (VF) replicas over seven days (Scale bars are 1000 μm and 250 μm for upper and lower rows). VF-shaped constructs encapsulating 3T3 fibroblasts within 5% GelMA were subjected to daily airflow-induced oscillation. Green fluorescence indicates viable cells, while red fluorescence indicates dead cells. (ii) Cell viability (%) shows an initial high viability followed by a gradual decline due to cumulative mechanical stimulation.
